# P-177. Contemporary Clinical Characteristics and Outcomes of Leprosy— a Multicenter Network Analysis

**DOI:** 10.1093/ofid/ofae631.382

**Published:** 2025-01-29

**Authors:** Samantha R Kaplan, Andrés F Henao Martínez, Masson Bliss, Christian Olivo Freites, Nelson I Agudelo Higuita, Luis A Marcos, Edgar A Ramirez-Garcia, Martin Casapia, Amir Mohareb, Charlotte Avanzi, Daniel B Chastain, Carlos Franco-Paredes

**Affiliations:** University of Colorado Anschutz, Denver, Colorado; University of Colorado Anschutz Medical Campus, Aurora, Colorado; University of Colorado Anschutz Medical Campus, Aurora, Colorado; Ryan Health, New York, New York; University of Oklahoma Health Sciences Center, Oklahoma City, OK; Renaissance School of Medicine at Stony Brook University, Stony Brook, New York; National University of the Peruvian Amazon-UNAP, Iquitos, Loreto, Peru; Universidad Nacional de la Amazonia Peruana, Iquitos, Loreto, Peru; Massachusetts General Hospital Division of Infectious Diseases, Boston, MA; Colorado State University, Fort Collins, Colorado; University of Georgia College of Pharmacy, Albany, GA; Hospital Infantil de Mexico, Mexico and Instituto Conmemorativo de la Salud, Panama, Denver, CO

## Abstract

**Background:**

Leprosy, also known as Hansen's disease, is a chronic infectious disease caused primarily by *Mycobacterium leprae*, endemic to tropical countries. In 2022, WHO registered 165,459 cases of leprosy. We lack more contemporary clinical descriptions and outcomes of the disease. Understanding its clinical characteristics is essential for improving diagnosis, treatment, and patient outcomes. We aim to describe the clinical manifestations and outcomes associated with leprosy using a "real-world" database.

US Map of the proportion of captured cases by region

**Figure:**
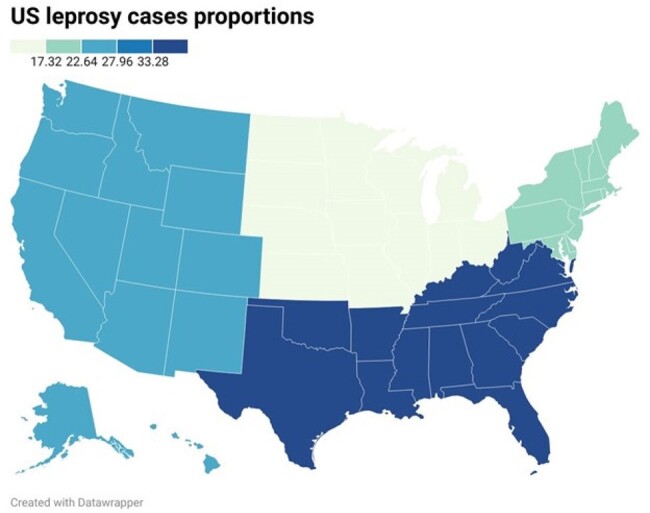

**Methods:**

We queried TriNetX, a global research network database (https://trinetx.com/), to identify patients with Leprosy by ICD-10 code. We captured demographics, comorbidities, clinical manifestations, treatments, and outcomes within 1 year.

Clinical characteristics and outcomes of leprosy patients by region

**Figure:**
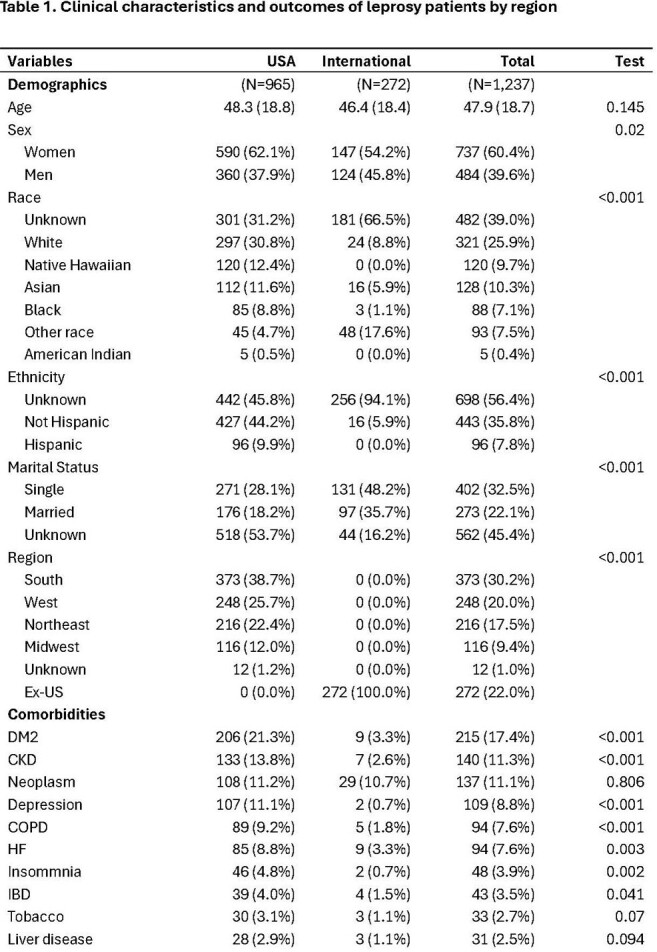

**Results:**

We captured 1,237 patients, with 965 (78%) originating from the US health system. The mean age was 48, and most were women (60.4%). US patients were predominantly from the South and West (Figure 1) and were predominantly White, Native Hawaiian, or Asian (table 1). The most common clinical manifestations included neuralgia, rash, neuropathy, and malaise. Blindness, corneal abrasions, and burns were present among 2-3% of US patients. Orchitis, iritis, and the Lucio phenomena were relatively uncommon. Rifampin, dapsone, and minocycline were most commonly used. However, significant geographical differences were noted in treatment regimens. Notably, clofazimine use was minimal. Steroids were used in 60% of cases, with a higher prevalence in US-based patients. Other immunosuppressants were uncommon, although methotrexate use was more frequent internationally. TB co-infection was 2%. The overall 1-year mortality was 24.3%.

Clinical characteristics and outcomes of leprosy patients by region

**Figure:**
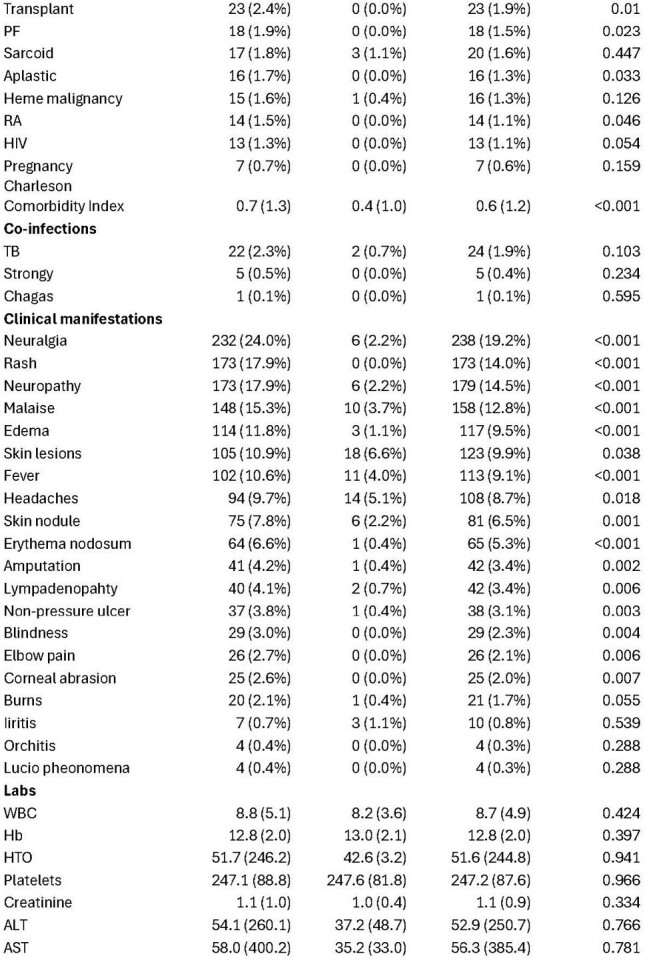

**Conclusion:**

Women from the southern and western US were the most significantly affected demographic groups. Severe complications like blindness, corneal abrasions, and burns, though less frequent, underscore the potential impact on quality of life. The presence of conditions like TB co-infection and the use of steroids in treatment point to the need for comprehensive preventive care strategies. With an overall 1-year mortality of 24.3%, our findings stress the importance of early diagnosis and treatment to improve outcomes and reduce mortality rates.

Clinical characteristics and outcomes of leprosy patients by region

**Figure:**
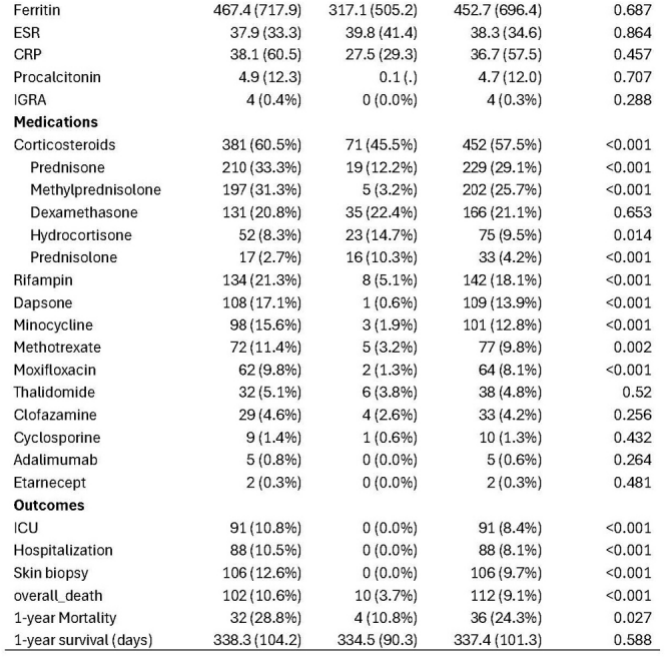

**Disclosures:**

**All Authors**: No reported disclosures

